# Skittle: A 2-Dimensional Genome Visualization Tool

**DOI:** 10.1186/1471-2105-10-452

**Published:** 2009-12-30

**Authors:** Josiah D Seaman, John C Sanford

**Affiliations:** 1 FMS Foundation, 7160 Stone Hill Rd., Livonia, NY 14487, USA; 2 Dept. Hort. Sci., NYSAES, Cornell University, Geneva, NY 14456, USA

## Abstract

**Background:**

It is increasingly evident that there are multiple and overlapping patterns within the genome, and that these patterns contain different types of information - regarding both genome function and genome history. In order to discover additional genomic patterns which may have biological significance, novel strategies are required. To partially address this need, we introduce a new data visualization tool entitled Skittle.

**Results:**

This program first creates a 2-dimensional nucleotide display by assigning four colors to the four nucleotides, and then text-wraps to a user adjustable width. This nucleotide display is accompanied by a "repeat map" which comprehensively displays all local repeating units, based upon analysis of all possible local alignments. Skittle includes a smooth-zooming interface which allows the user to analyze genomic patterns at any scale.

Skittle is especially useful in identifying and analyzing tandem repeats, including repeats not normally detectable by other methods. However, Skittle is also more generally useful for analysis of any genomic data, allowing users to correlate published annotations and observable visual patterns, and allowing for sequence and construct quality control.

**Conclusions:**

Preliminary observations using Skittle reveal intriguing genomic patterns not otherwise obvious, including structured variations inside tandem repeats. The striking visual patterns revealed by Skittle appear to be useful for hypothesis development, and have already led the authors to theorize that imperfect tandem repeats could act as information carriers, and may form tertiary structures within the interphase nucleus.

## Background

Recent discoveries are changing our appreciation of the complexity of genomic information. This includes strong conservation in non-coding regions, large scale transcription from areas previously considered to be non-functional DNA, bidirectional promoters, and alternative splicing patterns [1]. In addition, several unique coding patterns have been discovered that transcend the level of the classic gene, including a nucleosome binding code and a histone modification code [2]. In order to identify new encoding patterns with potential biological significance, novel strategies are required. It was for this reason that Skittle was developed.

It is well known that the human mind excels at pattern recognition (*e.g*., near-instantaneous face recognition), but the mind needs graphical help when dealing with large amounts of numerical data, and there is currently a lack of tools available for genomic visualization in general. Several abstract tools do exist, but we felt a need for the introduction of a simple tool that is optimized for the visualization of genomic data specifically. The primary goal in the development of Skittle was to give the user the simplest possible way to visually examine raw genomic data.

Skittle was not designed to compete with the data heavy, directed search algorithms already in existence. These are excellent at detecting repeats and other genomic motifs, but most lack a high quality visualization feature. Skittle was designed around the need for visualization, not automated data generation.

A very significant aspect of Skittle is frequency analysis of repeating patterns. Frequency analysis of DNA sequences has already revealed important discoveries. Patterns identified so far include certain 3-, 10-, 11-, 200-, and 400-base-pair motifs [3]. Some of these patterns have been associated with known genetic factors. For example, a 3-base-pair repeating pattern has been found to be characteristic of exonic regions [4,5], and a 10-11-base-pair motif has been found to be characteristic of DNA prone to supercoiling [6]. We expect a large part of future genomic analyses to involve the recognition and mapping of these and other multi-dimensional patterns.

### Background on Visualization Tools

SpectroFish [7] is in some ways similar to Skittle, but focuses on Fourier analysis. While it is adept at identifying open reading frames, it lacks a direct visualization of the raw data and thus users miss other interesting features (e.g., nucleotide bias). Also, the algorithm glosses over indels in repeat regions, while Skittle often reveals interesting indel patterns. DNA Rainbow [8] is an internet project that also employed the idea of representing the four nucleotides with colors, similar to our nucleotide view. The view was fixed at a width of 3500 nucleotides. This meant that, while they properly visualized the changing nucleotide bias, the only repeats that were recognizable were factors of 3500. Skittle expands upon this functionality by implementing a variable width so that all tandem repeats can be visualized.

Tandem Repeat Finder [9] was written to detect tandem repeats in genomic data. This program generates useful data that is not unlike the calculations upon which Skittle is based. Its main limitation is that there is no method of data visualization built into it. The user is presented with a list of sequences, the number of tandem occurrences, and various statistics associated with each repeat. This is useful information, but limited because the user cannot intuitively see many internal structural features of repeats that are apparent using Skittle.

REPvis [10] was another important milestone in the visualization of genomic data, but it focuses primarily on the physical location of distributed repeats. This specialization makes REPvis an efficient tool for this one task. Skittle's more broad approach is not optimized for distributed repeats and thus does not mask other features like nucleotide bias and variation inside repeats, which might be informative. In fact, Skittle reveals very interesting indel and point substitution patterns within tandem repeats which we feel are informative.

Skittle circumvents several of the limitations of these prior methods by using a straightforward, two-dimensional, four-color visualization that stays as close to the raw data as possible. It allows users to quickly analyze any FASTA sequence data and to readily identify and characterize tandem repeats, sequence structure, and nucleotide bias. This is coupled with a Repeat Map that detects and visualizes all similar sequences inside of the view window. These two features have never been combined in a simple yet powerful way.

## Implementation

Skittle consists of a collection of modular components written in C++. Each module is designed to visualize a different aspect of the sequence. Each module builds on the ones before it by leveraging previously computed data. The program uses three critical variables which can be controlled by the user to navigate the genome sequence, regardless of the exact module being used. *Start position *is the first nucleotide index that is displayed and is used to scroll through the sequence. *Width *is listed in nucleotides per line and handles the two dimensional component of the program, adjusting the visual representation of the data. Finally, *scale *allows the user to 'zoom out' to get a big picture view of the sequence. In general, *scale *is the number of nucleotides that a single pixel represents. In the following descriptions, program variables will be listed in italics for clarity.

### Annotation Display

**Figure 1:**
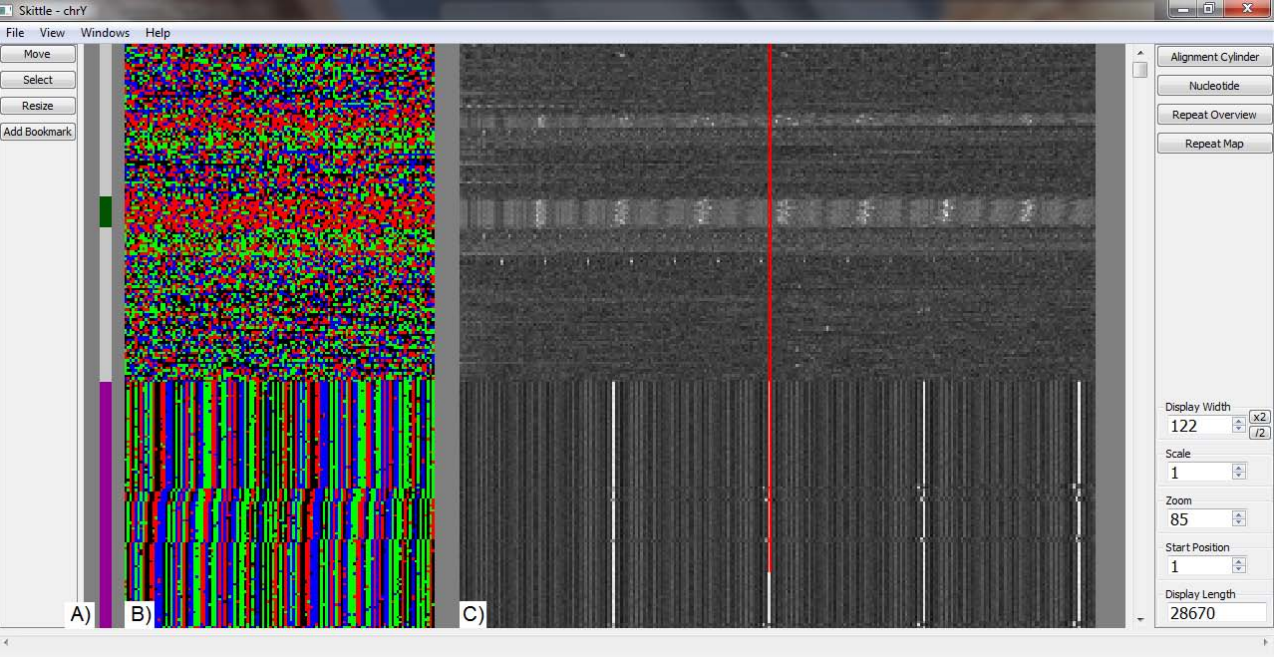
**Skittle user interface**. This shows the first 28,670 nucleotides of the Human Y chromosome. A) The Annotation Display is a single bar that shows annotation entries alongside the Nucleotide Display. The two annotations shown here are user notes created using Skittle. B) The Skittle Nucleotide View, with black = A, red = C, green = G, and blue = T. Unsequenced gaps (represented by "N" in the data file) are in gray. A high copy repeat of length 61 can be seen in the lower portion. Since the width of the nucleotide display is set to 122, two repeats appear on each line. C) The Skittle Repeat Map uses each row of the Nucleotide Display as a search string. The vertical red bar is a reference to the Width setting in the Nucleotide View in this case, width 122. The Repeat Map allows the user to see other repeats that are not currently aligned, such as the C-rich (red) repeat in the middle of the view window.

For any given DNA sequence Skittle can import and read previously developed Gene Transfer Format (GTF) [11] annotation files from other sources, and can display this annotation informationalongside the Skittle display modules. This provides a known frame of reference for the three primary Skittle display modules. The Skittle Annotation Display is a single bar with colored markers that correspond to the imported annotation entries (Figure 1A). The start and end indexes are extracted from the file and used to create track entries. Track entries are each assigned a random color to make differentiating adjacent entries easier. The track text that is associated with each annotation entry can be retrieved by simply selecting the entry. This allows users to easily place the visual information they are observing within the correct genomic context. This frame-of-reference feature has been used for most of the observations listed in this paper. This feature enables users to correlate previous research data with the visual patterns seen in Skittle.

### Nucleotide Display

The simplest and least abstract Skittle module is the Nucleotide Display. Instead of displaying letters, each nucleotide is represented by a different colored pixel (black = adenine, red = cytosine, green = guanine, and blue = thymine). The point at which the nucleotide string wraps around to the next line is referred to as the *width*. *Width *is determined by the user and is the essential variable for visualizing tandem repeats. When the *width *is set to any multiple of a basic repeating unit, the repeats appear as a series of vertical bars (Figure 1B). This simple visualization tool immediately reveals many major repeats.

The typical view window is around 200 × 200 pixels or 40,000 bp, which is only a small fraction of a human chromosome. Since the goal of Skittle is to allow the user to look at *all *of their data, the program also incorporates a *scale *factor that allows the user to zoom out indefinitely. Looking at individual nucleotides could be equated to the street view on a satellite image, but the user can also zoom out to see the shapes of continents or in this case, chromosome-level features. The Nucleotide Display implements *scale *by averaging the color values of a number of nucleotides equal to *scale *into a single pixel. So at *scale *= 54 each colored pixel represents 54 nucleotides. This is essentially a visualization of the changing nucleotide bias at a given *scale*.

### Repeat Map

More subtle genomic patterns are revealed using the Repeat Map. This more advanced visualization tool runs parallel to the Nucleotide Display (Figure 1C). The Repeat Map can detect the lowest level of tandem repetition (two copies of one sequence) - which is displayed as a single light-colored pixel. Not only do repeats become more readily visible, but their characteristics and internal variation (differences in length and percent identity between repeating units) can be readily seen. Many patterns too subtle for detection using the Nucleotide Display are clearly revealed using the Repeat Map.

When searching for tandem repeats the program is essentially looking for an offset where the sequence has a greater than random similarity to a sequence at a fixed distance downstream. This distance is called the *offset*. Since we observe regular repeating sequences inside of genomes we can refer to the *offset *as a periodicity or frequency. The frequency is the number of base pairs separating similar sequences. Therefore, when searching for tandem repeats, we are essentially looking for any dominant frequency pattern. Frequency does not mean the number of times something occurs in the genome, but rather the local periodicity of the sequence. Using this simple analogy, we can create a map of the local alignment space. As a clarification, this paper uses the term 'local' and 'local alignment' simply to refer to a sequence match in the local area around a specific start position, rather than to refer to any specific algorithm.

The purpose of the Repeat Map is to detect similar pairs of neighboring sub-strings within a sequence. The *width *in Nucleotide Display becomes the *sample size *in the Repeat Map. This means that each row within the Nucleotide Display is a *reference string*, with a length equal to the *sample size*. The Repeat Map's x-axis is the range of *offset*. The *target string *is computed by adding *offset *to the start of the *reference string*. *Score *is computed by counting the number of exact matches between the *reference string *and the *target string*. By dividing *score *by *sample size*, the program calculates percent identity. The gray scale value of each pixel in the Repeat Map depends on the percentage identity between the *reference string *and the *target string*. Gray scale values range from white for near-perfect similarity, through various shades of gray, to black for zero similarity.

At *scale *= 1 the range of offset is 1 to 250 nucleotides downstream. The Repeat Map is capable of zooming out to higher scales. So at *scale *= 1000, each pixel in the Nucleotide Display represents 1,000 nucleotides, so the actual offset range of the two strings being contrasted would be 1,000 to 250,000 nucleotides. At *scales *greater than one, Skittle takes the color averaged string (see Nucleotide Display description) and finds the correlation coefficient of the two strings. This looks largely the same as a base pair by base pair comparison except for two differences. The first difference is speed: correlation is much faster at larger scales. Performing the computation on the color compressed string means that correlation is always applied to 250 pixels in constant time, regardless of the scale. If the number of comparisons is multiplied by the scale, then the Repeat Map would quickly become unusable at large scales. The second difference is that mismatches are scaled appropriately to the size of the repeat. For example, a deletion of 40 nucleotides in a 4,000 bp monomer is still only a 1% shift. The gray scale coloring remains largely the same with white representing a correlation coefficient of 1.0 and a correlation 0.0 (random) represented by 50% gray. This allows the user to spot larger features, such as segmental duplications, as easily as one can find short tandem repeats.

### Repeat Overview

**Figure 2:**
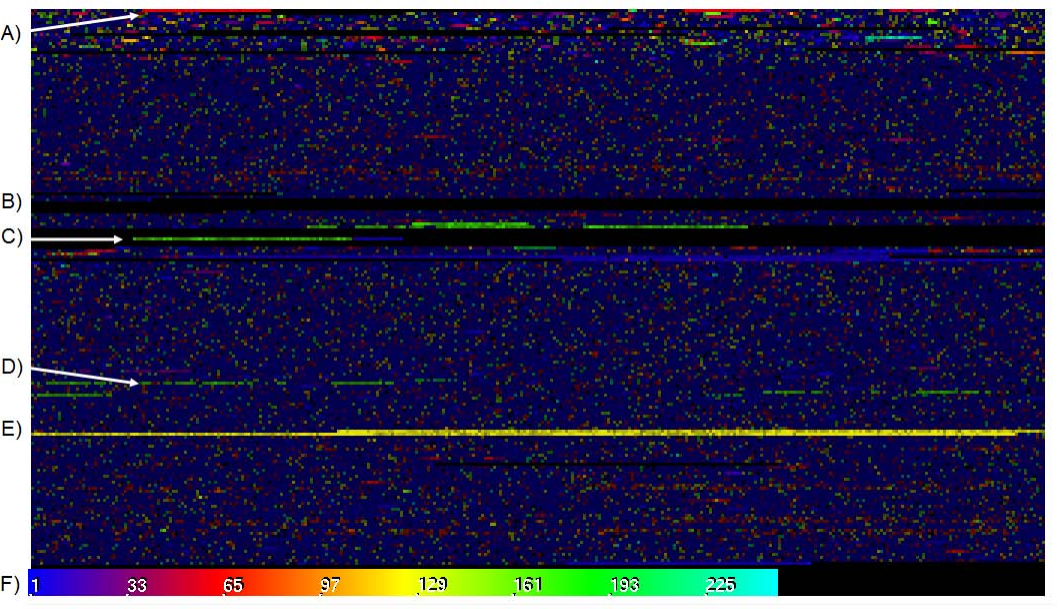
**Repeat Overview of Human Y Chromosome**. This visualization of the first half of the Y chromosome is colored according to the best local alignment score. Chromosome Y was selected for the diversity of repeats. The second half of the Y chromosome has only a very small section near the end that has been sequenced so it is not included in this view. A) The bright red streak is the same 61-mer repeat seen in Figure 1B. B) The pure black streaks are regions of unsequenced N's. C) The green areas in the middle are 171-mer alpha satellite near the centromeres. D) A second region of alpha satellite repeat (with lower percent similarity) separated from the centromere starting around nucleotide #18,293,073 (March 2006 assembly). This is actually part of a large scale palindrome where the reverse compliment of the alpha satellite starts at # 18,822,065. E) The bright yellow region is a very large, 125-mer tandem repeat that comprises a quarter million nucleotides. F) Colors correspond to the frequency spectrum ranging from 1 - 250 nucleotide offsets. Brightness is based on the degree of local self-similarity, with brighter colors corresponding to better self-similarity.

The Repeat Overview allows the user to visualize in a single window the dominant tandem repeat patterns within much larger portions of a chromosome, or even within the entire chromosome (Figure 2). The Repeat Overview was designed as an overview of the information contained in the Repeat Map. Each row of the Repeat Map represents a local area of the sequence. The Repeat Overview takes each row of the Repeat Map and reduces it to a single pixel by taking only the *offset *with the highest *score *(best match).

The raw data for Repeat Overview is a list of ideal offsets, based on the Repeat Map, and their corresponding similarity *score*. Tandem repeats will appear as a contiguous set of data points where the ideal alignment is consistently the same and has a high *score*. Indels will be minor variations in the alignment, and unique DNA will be regions that have a low similarity *score *and no clear alignment consensus. The Repeat Overview is the highest degree of information density in Skittle because a short list of values can tell the user the monomer length, nucleotide coverage, insertions/deletions, and distribution of every tandem repeat in an entire chromosome. Obviously it's easy to get overwhelmed by this amount of information, which makes data visualization an important part of the process.

In the Repeat Map, the program has two pieces of data - the local offset and the percent similarity at that offset. The percent similarity (or *score*) for any given pixel is represented by its lightness, ranging from black to white (white being 100% identity).. The Repeat Overview also uses brightness to represent how well the sequence matches. The ideal offset is represented by the color hue of the pixel, allowing it to compress the information from an entire line into a single pixel. This means that an equivalent 250 × 180 pixel Repeat Map will compress into only 180 pixels in the Repeat Overview. To represent the local alignment using color the program maps the range of possible alignments (1-250 by default) to a color spectrum. This mapping can be seen in the labeled spectrum (Figure 2F). The ideal offset determines the hue, and the percent similarity determines the brightness. In this way, the user can see the information garnered from billions of comparisons communicated through a single colored image.

The Repeat Overview reveals strong tandem repeats, such as sub-telomeric repeats and alpha-satellite DNA, as brightly colored areas. Duller colors reflect lower levels of local self-similarity. The specific hue of the color is used to represent the dominant frequency of a repetitive region (i.e., blue and purple pixels reflect shorter repeats). In areas of the genome where there are no substantial repetitive DNA sequences (very dull colors), the highest alignment score is rarely above 30%, while areas containing strong tandem repeats (bright colors) often have 90% or higher internal alignment scores. The end result is that highly self-similar areas show up as regions of bright colors, and the color's hue indicates the size of the dominant repeating element. Areas with no significant repeats appear a dark blue color.

### Alignment Cylinder

While the Nucleotide Display is useful, it has two fundamental shortcomings. First, the fixed width means that the user can not see repeats of different frequency at the same time. Second, the edges of the Nucleotide Display imply a disjunction, whereas DNA is actually a continuous string. The continuity issue is solved if we connect the trailing edge of the Nucleotide Display to the leading edge on the next line. In 3D space, this folds a flat plane into a cylinder, where the DNA sequence makes a continuous spiral around the circumference of the cylinder. The second step is to allow the circumference of the cylinder to change, resulting in a variable width cylinder similar in appearance to a spindle that has been cut on a lathe. This allows the program to display repeats of different widths simultaneously.

**Figure 3:**
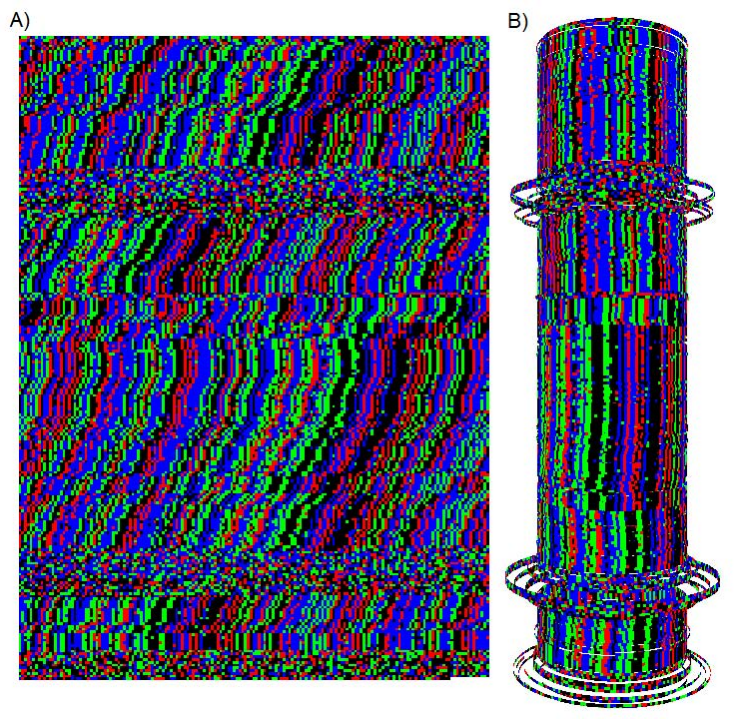
**Alignment Cylinder**. A genomic sequence from Human Y chromosome in the nucleotide view (A) is shown alongside a hypothetical 3D cylindrical architecture (B). The example sequence is human alpha satellite that is interrupted by a few pieces of non-alignable DNA. The primary repeat has a circumference of 171 bp and tiny variations in the cylinder's diameter negate most of the indels so that the repeat aligns vertically. This creates a continuous alignment in 3D space without the introduction of any gaps or other artificial constructs. The areas of non-alignable DNA are represented by loose, unorganized loops since they have no obvious preferred position.

The Alignment Cylinder is a new approach to visualizing sequence alignment (Figure 3). The underlying structure is a gradually spiraling string of nucleotides with an adjustable circumference. The program identifies the best local alignment using a similar algorithm to the Repeat Overview and smoothly adjusts the local circumference based on the best alignment. Cylinder Display needs to be more sensitive than Repeat Overview because the circumference can be adjusted at every nucleotide position. It does this by identifying 'seeds' where a perfect match of eight nucleotides can be found downstream. The program then uses the seeds to establish a local *consensus width*. Next, it checks adjacent nucleotides near the *consensus width *using a low threshold. In this way, the starting seeds are expanded into larger patterns that follow the changes in width of the repeating sequence.

Using a high threshold to start new patterns and a lower threshold to expand existing patterns is analogous to Canny Edge Detection [12] in the field of image computation or ice crystal formation in chemistry. This method is more sensitive to small changes in alignment than the Repeat Map, but it is also more computationally intensive. The display is useful for looking at different tandem repeats simultaneously as well as visually smoothing out sequences with many indels in them. The displayed cylinder can be graphically rotated to observe all aspects of the alignment.

## Results

### 1) Detecting Patterns of Nucleotide Composition

Using the Nucleotide Display, it is immediately obvious that most genomes are made up of regions strongly dominated by specific nucleotides, and this is seen at all scales. Taking a random window from the human genome, we can see alternating regions which are on the scale of thousands of nucleotides, and are predominantly either AT-rich or GC-rich, and these often have sharply defined borders [Additional file 1A]. This stands in contrast to what we observe with randomly generated sequences [Additional file 1B], which do not have any visually obvious patterns of nucleotide composition. This phenomenon is also observed at much larger scales, and when Skittle is "zoomed out" we see visual patterns on the scale of millions of nucleotides, which we observe to correspond directly to recently mapped human isochores [13].

### 2) Detecting Un-annotated Repeats

Skittle is an easy way to visually inspect automatically generated data. Visual inspection allows a researcher to discover repeats that the other computer programs may have missed. As an example, Skittle was used to visualize the repeat masked genome sequence downloaded from UCSC Genome Browser [14]. Masked sequences are useful when a researcher wants to exclude repeating DNA from their study. The sequence was masked using two programs. First, RepeatMasker marked all subsequences that matched the known repeats in its library. Second, Tandem Repeats Finder was used to identify all tandem repeats with a periodicity of 12 bp or less. Under this methodology any tandem repeats with a monomer length of more than 12 bp which also didn't occur in the sequence library would be missed, yet would be easily detected by Skittle.

**Figure 4:**
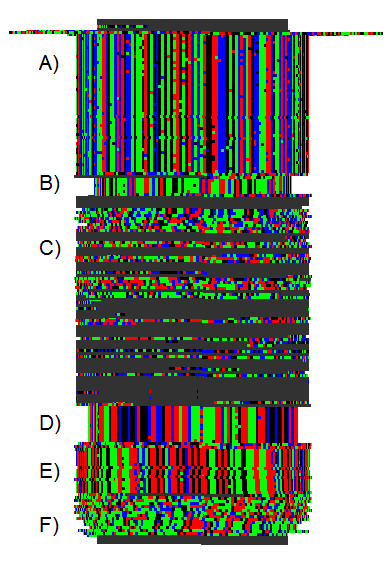
**Unmasked Repeats**. Cylinder Display showing large tandem repeats still remaining recognizable by Skittle, in the Repeat Masked Sequence (Feb. 2009). This section of Human Chromosome Y still displays many large repeats because they were outside of the scope of the masking program settings. A) 61 bp repeat also shown in Figure 1. B) 26 bp repeat. C) A region of unique DNA and sequence masked by N's (gray). D) 55 bp repeat. E) 49 bp repeat. F) 68/81 bp repeat. The alignment on this final repeat is not very clear from the sequence but it is still visible as repeating DNA when visualized in Skittle.

Using Skittle, one can identify sequences that are missed by an automated method and make an informed decision about whether data is suitable for the planned research. Figure 4 shows several examples of repeats that are obvious to the human eye when visualized in Skittle but were missed in the repeat masked sequence because they fell outside of the program parameters. There are several other repeat-finding algorithms available for public use. The data from any of these can be combined in a GTF file and displayed alongside the Skittle Nucleotide Display (Figure 1A) or masked sequences can be visualized to ensure data quality. We find this a useful feature for visualizing the large amounts of numerical data that other search algorithms can produce.

### 3) Analysis of the Human 171-base-pair Centromere Repeat in Both Two and Three Dimensions

The human centromere is dominated by alpha-satellite DNA with an average repeat width of 171 base pairs. This is the most visually obvious large-scale frequency pattern within most human chromosomes (Figure 2C). On the Repeat Overview, this repeat shows up as bright green regions bracketing the unsequenced (black) centromeric regions. Alpha-satellite DNA also tends to have a very high indel and substitution frequency, making the internal percent identity much lower than other "clean" looking tandem repeats (Figure 3A).

When we self-align this sequence to create a 3-dimensional coil structure, we see that the entire structure becomes visually more coherent (Figure 3B). Deletions become indentations in the cylinder and insertions become outer loops on the surface of the cylinder. This basic architecture effectively smoothes out indels, such that sequences with many phase shifts align better in three-dimensional space than they do in the two-dimensional Nucleotide Display.

### 4) Analysis of Alu Repeats

Despite many repeats within the human genome which are visually striking in the nucleotide display, such as alpha satellite DNA, the most common repeat in the human genome is actually a dispersed 135-bp structure which is not even visible in the Nucleotide Display, but is only seen in the Repeat Map. This dispersed repeat is visible as a recurrent series of single pixels throughout the Repeat Map for all human chromosomes, at or very near the offset distance of 135. This hyper-abundant repeat is caused by an internal repeat within the Alu elements that saturate the human genome. By making a histogram of individual frequency alignments for an entire chromosome [Additional file 2], it can be seen that this 135-mer repeat is the dominant repeat of the sequenced human genome (excluding a profusion of diverse very short repeats). This dispersed repeat appears to be the strongest repeat signal in the human genome, but will almost certainly be displaced by the 171-mer centromeric alpha satellite repeat (which is also visible in this figure), because human centromeres have for the most part not yet been sequenced.

In order to confirm the identity of this frequency band, the annotation track from RepeatMasker was imported and displayed next to the Repeat Map. At the nucleotide level, the 135-mer pixels very consistently corresponded with individual Alu annotations [Additional file 3]. This dispersed repeat is caused by a string of approximately 70 nucleotides within the Alu element, followed by a unique spacer of about 65 nucleotides, and then the original 70 nucleotide string is repeated. The beginnings of the two repeat units are spaced 70 + 65 = 135 nucleotides apart, which determine the visible frequency band. Alu has a high amount of variation between individual elements so the exact offset of the internal repeat tends to vary from 133 to 139.

### 5) Analysis of Repeats at All Scales

**Figure 5:**
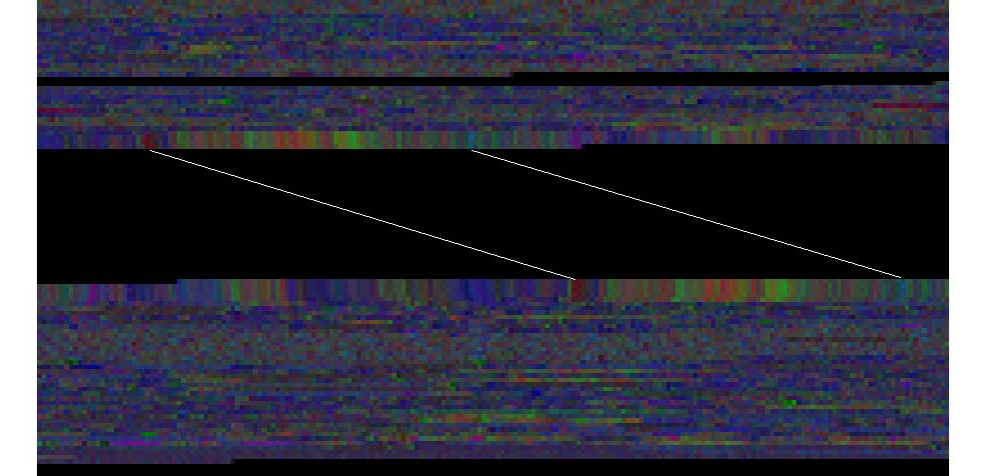
**Segmental Duplication**. A segmental duplication found on the human Y chromosome at positions 8,590,000-11,000,000. The monomer is approximately 20,344 nucleotides, with 9 visible copies spanning an unsequenced region (black). The mega-repeat probably extends through the unsequenced region, with the sequencing gap resulting from alignment difficulties within this repetitive region. Note that the upper and lower sections are not in phase with each other (the diagonal line shows where the first section lines up with the beginning of the second section). This suggests that the length allotted to the unsequenced region may not be correct.

Tandem repeats are generally divided into categories based on the size of the repeating unit. Using Skittle, we have observed that there are examples of tandem repeats at all scales. For example, the *D. melanogaster *genome contains a large number of tandem repeats in the 300-400-mer range [Additional file 4]. It is also common to see the same repeat show up in many places in the genome [Additional file 5]. The largest-scale repeat detected thus far with Skittle is on human chromosome Y. It is a c.a. 20,300-nucleotide repeat with a total of 9 tandem copies bracketing a large un-sequenced (black) region (Figure 5). The recognizable visual pattern shown in the figure is actually caused by an obvious periodicity in the GC-rich (green/red) regions and AT-rich (black/blue) regions.

### 6) Analysis of Nucleotide Covariance

**Figure 6:**
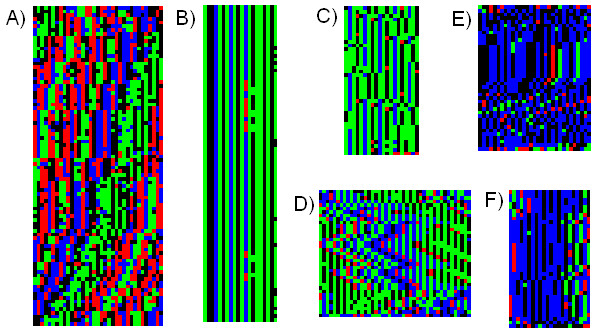
**Repeat motif examples from human chromosome 19**. A) 35-bp repeat that frequently alternates with 171-bp centromeric alpha-satellite DNA. B) This repeat has no indels and point substitutions only occur in 3 of the 20 columns. C) It is common to see similar motifs contained in other repeats. This repeat shares the sequence GAGT with Figure 6B, but they are obviously different sequences with different patterns of variation. This sequence has irregularly spaced insertions of 4 nucleotides along with C often substituted for A. D) A repeat with many indels in multiples of 2. The various repeats align at 42, 44, and 46 bp. E and F) Two examples of a common repeat motif that occurs throughout the human genome. They are dominated by an internal AT repeat and generally have no single clear alignment.

**Figure 7:**
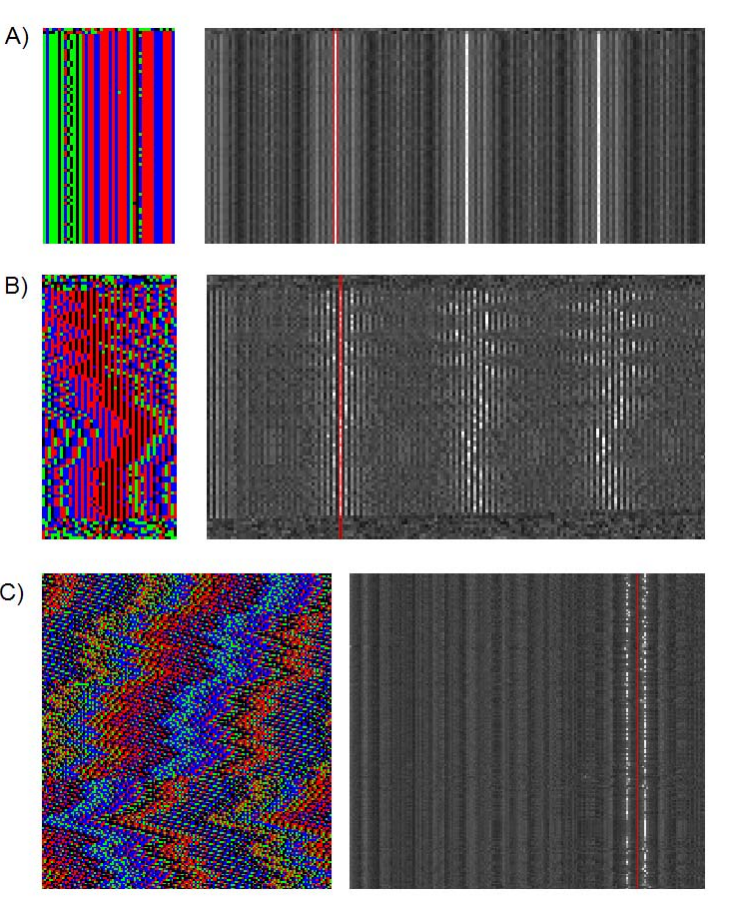
**Examples of Structured Variation**. The Nucleotide Display is on the left with their corresponding Repeat Maps on the right. A) Nucleotide covariance within a tandem repeat on human chromosome Y: 392,702-397,700 (March 2006 assembly). We can refer to each monomer position as a column labeled 1-44, starting with column 1 on the left. There are only four columns with any significant amount of variation (columns 8-10, and 33). The consensus in column 33 appears to be T (blue) but 26 out of the 27 point substitutions in that column are Gs (green). Columns 8-10 appear to have a consensus of TGA but all variation in these columns occurs when all three positions are simultaneously replaced with CAG 34 times over the course of the repeat. B) A complex tandem repeat pattern on human chromosome 19, with monomer widths of 41, 43, 45, 47, or 49. This pattern appears to have a strong dimer component running throughout. Notice that all shifts in the repeating pattern are in multiples of two, yet the pattern itself actually has an odd width around 45 nucleotides. This kind of indel offset consensus is a fairly common occurrence and can come in sets of 3 or more nucleotides as well. C) Shifting pattern in rice chromosome 12 centromere. This repeat has a dual frequency spacing of 155/165. The Repeat Map clearly shows two bands at these lengths. The nucleotide display is set to width 161. The overall picture is a zig-zag pattern which might arise via a series of systematic insertions or deletions. When the repeating monomer is 155 nucleotides long, the vertical bands slant to the right. When the monomer is 165 nucleotides long, it slants to the left.

Tandem repeats are seldom perfect repeats. Often, repeat units are variations on a repeating theme (Figures 6 and 7). This variation is not always random, and can be systematic in the case of nucleotide covariance (when one nucleotide deviates from the theme, other nucleotides also vary in a predictable manner). An example of this type of covariance is seen in a tandem repeat on human chromosome Y that shows a very high degree of covariance between nucleotide positions (Figure 7A).

### 7) Analysis of Dual Frequency Repeats

Tandem repeats sometimes display a dual-frequency motif. The rice *(Oryza sativa *L. ssp. *japonica*) genome contains a very large repeat near the centromere with repeat frequencies of 155 and 165 nucleotides (Figure 7C). Non-periodic deletions totaling ten base pairs per repeat copy occur throughout this region, forming two independent frequency signatures. This is not simply a "repeat of repeats" as there is no other higher order band, neither is this a simple repeat. This suggests that the variation inside of dual-frequency repeats represents systematic structure that is non-periodic in nature. This illustrates the power of Skittle in uncovering complex internal structures within tandem repeats.

### 8) Analysis of Alternating Repeats

Another common genomic pattern is caused by the periodic alternation of dissimilar repeats. An excellent example of this phenomenon is on human chromosome 19, just after the unsequenced centromere region [Additional files 6 &7]. Here one can see a clear alternation between centromeric alpha satellite DNA and a repeat of width 35 (Figure 6A). This pattern alternates five different times. More complicated patterns of alternating repeats can be observed throughout the genome. For example, near the centromere on human chromosome X there is a long stretch that alternates between 5 different sequences [Additional file 8].

### 9) Analysis of 3-mer Patterns Associated with Protein-coding Regions

While browsing genomes using Skittle we were surprised to often come across areas of the genome that were not tandem repeats, but which displayed a 3-mer visual texture in the Repeat Map [Additional file 9]. Using annotation tracks, we confirmed that these sequences correlated very closely to protein coding regions. This is a basic signature of protein coding regions already documented in the literature [3-5]. The basic 3-mer frequency pattern visualized in Skittle has previously been useful for identifying exons and even indicating reading frame and direction of the exon [7]. The 3-mer pattern is so systematic in *E. coli *that when we zoom out to see the entire genome, it is the only distinct genomic feature, and unifies the genome as a single frequency image [Additional file 10]. This illustrates the utility of Skittle, in that users can find biologically meaningful patterns, supported by literature, even when they aren't specifically looking for them.

## Discussion

Most current methods of analyzing repeats focus on the size and number of the repeating unit, but Skittle also allows the user to analyze variation within the whole repeat assembly. The program does not mask over internal variation. By not treating SNPs and indels as noise, we retain a significant amount of information that is filtered out by many other methodologies. In many cases, tools are designed to find predetermined patterns, and therefore ignore discordant data. In other cases, the more comprehensive tools may include all the data, but in a numerical form which may be difficult to fully process and interpret. The design of Skittle allows it to address both problems simultaneously. It creates a comprehensive analysis of the repeat information content of a genome, but with a graphical, rather than a numerical, representation.

Tandem repeats comprise a very significant part of higher genomes. The greater parts of the unsequenced centromeric and heterochromatic regions of higher genomes appear to be extensive tandem repeats [15,16]. In human, these regions may constitute 10% of the genome. Tandem repeats have historically been assumed to be "junk DNA", but it would be prudent for us to keep an open mind to unexpected types of functionality for tandem repeats [17,18]. If tandem repeats have any function at all, such function would not be expected to relate to protein production. A more likely function might involve structural information.

As we have been able to better visualize tandem repeats using Skittle, we have seen a surprising amount of internal complexity. Some of this complexity seems to be easily understood in terms of point mutations and indels, but a great deal of the complexity appears to provide an intriguing array of "puzzles" which invite further study. These puzzling patterns include co-varying deviations from a repeating theme, and internal patterns that are not simply "repeats within repeats". For lack of a better term we are referring to these patterns as *structured variation*.

If tandem repeats have any function, the "structured variation" described above could conceivably carry information. A perfect repeat cannot contain any information beyond the base sequence and copy number. However, a repeat with variation can contain considerably more information. Each of the three types of observable variation (substitutions, indels, and alternating repeats) has a direct analog in electronic information technology (amplitude modulation, phase modulation, and frequency modulation, respectively). One possibility is that a repeat sequence could have biological significance by affecting chromatin structure. Substitutions and indels inside repeats might provide structural nuance or act as binding sites.

DNA is a linear molecule but can take on various 3-dimensional configurations. There is growing interest in higher-level DNA architecture within the interphase nucleus, and 3-D structures have been proposed ranging from loops and local coils, up to whole chromosome domains, up to comprehensive whole-genome 3-D folding structures within the interphase nucleus [19]. Recent research has shown that homologous sequences of DNA spontaneously self-align, even in the absence of proteins [20]. Such self-alignment is apparently caused by very weak molecular attraction and is biologically important for processes such as homologous recombination, crossing over, and chromosomal alignment during meiosis. Given this knowledge, we reasoned that tandem repeats *in vivo *may actually form 3-dimensional structures similar to our Cylindrical Display, with the circumference of the coil being equal to a multiple of the repeat length (Figure 3 &4). Given that similar sequences self-align spontaneously, it is reasonable to assume this will routinely happen except when there is a counterforce to prevent it. The presence of nucleosomes would interfere with the formation of these loose coils, so such coils would be subject to the same chromatin regulating mechanisms as are genes. If such coiled structures were real, they would be very hard to detect, because they would be very delicate transient structures within the interphase nucleus, present only where the DNA was unpacked. They would not be expected to survive normal sample preparation for electron microscopy.

Interestingly, the self-adjusting cylinder alignment, which was designed to simply optimize local alignment as would be expected *in vivo*, causes a marked increase in the visual coherence of all complex tandem repeats. This suggests to us that such coherence might reflect a minimal energy state, and may reflect actual structure *in vivo*, and might even reflect an unknown biological function. Logically, such coils could change circumference in multiples of the repeat length and so might modulate local genomic architecture.

In addition to the specific examples of application we have described here, we envision Skittle (or similar derived programs) to serve in more generic genomic exploration. For example, it is possible to take a set of unassembled sequences for an organism for which limited genomic data is available, and visually analyze them with Skittle. The user can gain an intuitive understanding of their sequence data, including the amount of variation, degree of nucleotide bias, and the overall similarity of sequences. In addition, Skittle would allow this person to dump visually interesting snippets to a text file that could be used for further explorations (e.g., BLAST).

### Future Improvements

The program features presented in this paper (Nucleotide View, Repeat Map, Repeat Overview, and Alignment Cylinder), are the core modules of Skittle. They were specifically implemented to aid in research. There are many more Skittle features that have been prototyped, such as the repeat histogram [Additional file 2], which are currently under development. Additional functionality should add to the program's convenience but the core functionality already implemented represents the primary research work tools. We've specifically avoided implementing functions that duplicate pre-existing bioinformatics tools because Skittle is designed to work as part of a suite of tools. Future developments will be guided by how they can aid research. While this paper has primarily discussed tandem repeats, a major focus in the future will be visualizing the multitude of dispersed repeats.

While Skittle has immediate utility in identifying and characterizing tandem repeats, it is hoped that this program's multi-dimensionality can eventually have broader utility, being generally useful in characterizing the entire multi-dimensional genome. We seek to make this program as useful as possible to the genetics community, and welcome suggestions on how to make it more informative and more user-friendly. Additional information and a user's manual are available at http://www.DNAskittle.com.

## Conclusions

The data visualization program 'Skittle' is a new and useful tool for visualizing DNA sequence data. This program expands researcher's understanding of genomic patterns already described via numerical analysis, and helps researchers discover new genomic patterns not previously recognized. This tool is especially sensitive to any type of tandem repeat, and reveals structured variations which may suggest functionality for such repeats.

## Availability and Requirements

**Project name: **Skittle Genome Visualization

**Project home page: **http://sourceforge.net/projects/skittle/

**Operating system(s): **Windows (2000, XP, Vista, Win7) 32-bit, Linux, Mac OS X

**Programming language: **C++

**Other requirements: **Qt for Developing

**License: **GNU GPL

**Restrictions to use by non-academics: **none

## Authors' contributions

JDS drafted the original idea for genome visualization and was responsible for developing all the software content and the majority of the manuscript. JCS provided biological insight and developed the 3-D structural hypothesis for tandem repeats. All authors read and approved the final manuscript.

## Authors' Information

JDS has a bachelor's degree in computer science with a specialization in computer graphics. He recently started a Master's degree in Bioinformatics. JCS is a plant geneticist (Courtesy Associate Professor), at Cornell University.

## Supplementary Material

Additional file 1 **Comparison of a real DNA sequence and a randomly generated sequence**. A) This sequence is taken from Human Chromosome 10. It was chosen at random and contains no tandem repeats. Changing nucleotide bias can be seen, as reflected by regions which are AT-rich (black and blue) and regions which are GC-rich (green and red). B) The random sequence, in contrast, has no higher level patterns. This sequence was generated by a computer using a pseudo-random number generator. Click here for file

Additional file 2 **Histogram of the Repeat Overview pixel hue**. This histogram is taken from Human Chromosome 4 as a typical example. There is a very significant peak at 135 which is caused by the di-mer structure of the Alu repeat. Next to it is a much smaller peak centered at 171 caused by the centromere alpha-satellite repeat. Much of the centromere is unsequenced so the number of copies is under-represented. When selecting the 'best frequency' the Repeat Overview starts at offset 1 and all future values must beat the previous by 10%. This is to ensure that it does not display multiples of repeats and also causes the graph to favor shorter repeats. Click here for file

Additional file 3 **Alu repeats in Skittle**. Alu repeat annotations on Human chromosome 19 (left), shown next to the Nucleotide Display and Repeat Map. This annotation track is based upon RepeatMasker. As seen, this section is rich in Alu SINE repeat annotations, which correlate to pixels seen in the Repeat Map around 135 bp, and which reflect the internal repeat structure seen within most Alus. The Annotation Display appears as three lines in this image because annotation entries often overlap each other. Click here for file

Additional file 4 **Large repeats in *Drosophila *genome**. This tandem repeat is found in *Drosophila melanogaster *chromosome 2L. Its monomer length is 306 bp. The pattern starts at nucleotide #4,533,478. Click here for file

Additional file 5 **Reverse Complement Repeat**. *Drosophila melanogaster*: The top and bottom of this tandem repeat are actually reverse complementary sequences, making it a palindrome. Using Skittle, this repeat has been observed on multiple chromosomes. Chr2L: #21020819, Chr2R: none, Chr3L: #8013350, #20815000, #22737200, Chr3R: #17435800, #18277280, Chr4: none, ChrX: #2964690, #3621290, #11539710, #19251467, #21924980. Click here for file

Additional file 6 **Repeat Overview of Alternating Repeats**. Repeat Overview of a section of Chromosome 19 just below the unsequenced centromeres (black). A) The bright green pixels are alpha-satellite. The mixed dark purple and blue pixels represent areas with no tandem repeats alternating with the alpha-satellite repeat. B) The 35-mer repeat shows up in bright purple and orange. C) The darker colored region at the bottom is where unique, non-repeating DNA resumes after the centromere. Each pixel represents 170 nucleotides. Click here for file

Additional file 7 **Nucleotide Display of Alternating Repeats**. Alternating 171 and 35 repeats on Human Chromosome 19. These repeats often occur together. Click here for file

Additional file 8 **Repeat Map of Alternating Repeats**. Repeat Map showing another example of alternating repeats on Human Chromosome X. Each pixel row of the Repeat Map represents the local alignment of 1380 nucleotides. The map covers over 220,000 nucleotides. The repeats alternate between 5 different patterns: 171, 35, 171, 35, 171, 35, 171, 35, 193, 217, 183/217, 217, 171. This is immediately followed by the unsequenced centromere. Click here for file

Additional file 9 **Genes in *Drosophila***. Example of visible genes in *Drosophila melanogaster *chromosome 2L. Exons are accompanied by a faint, though recognizable, 3-mer frequency pattern caused by the codon bias of the organism. Potential exons can be visually identified using Skittle without any other knowledge. In the Human genome, exons are sometimes also visually recognizable because of a sudden change in nucleotide bias towards GC rich content (Additional File 1). Click here for file

Additional file 10 **Repeat Map of *E. coli***. The Repeat Map of the whole *Escherichia coli *genome reveals the 3-mer frequency pattern characteristic of exons. The majority of this genome sequence is protein coding, making it a better example than the human genome. The visible pixel pattern is dark, dark, light, which repeats every 3 pixels and is due to codon bias. This is an average of all local alignments ranging from offset 1 to 250, so it effectively negates the fact the individual exons exist in different reading frames. Click here for file
