# Supplementary figures and images for: Skittle: A 2-Dimensional Genome Visualization Tool
Source: BMC Bioinformatics. 2009 Dec 30;10:452. doi: 10.1186/1471-2105-10-452 (PMC2817707; doi:10.1186/1471-2105-10-452)

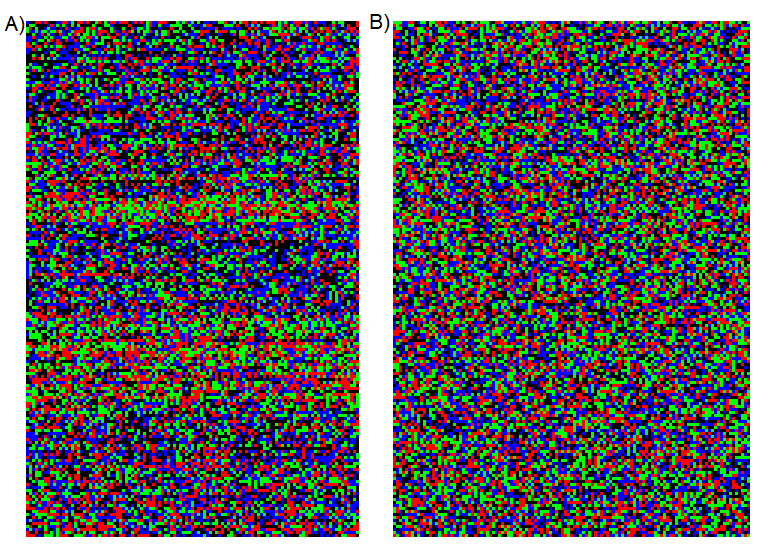

Supplement: Additional file 1 — Comparison of a real DNA sequence and a randomly generated sequence. A) This sequence is taken from Human Chromosome 10. It was chosen at random and contains no tandem repeats. Changing nucleotide bias can be seen, as reflected by regions which are AT-rich (black and blue) and regions which are GC-rich (green and red). B) The random sequence, in contrast, has no higher level patterns. This sequence was generated by a computer using a pseudo-random number generator. [file 1471-2105-10-452-S1.TIFF]

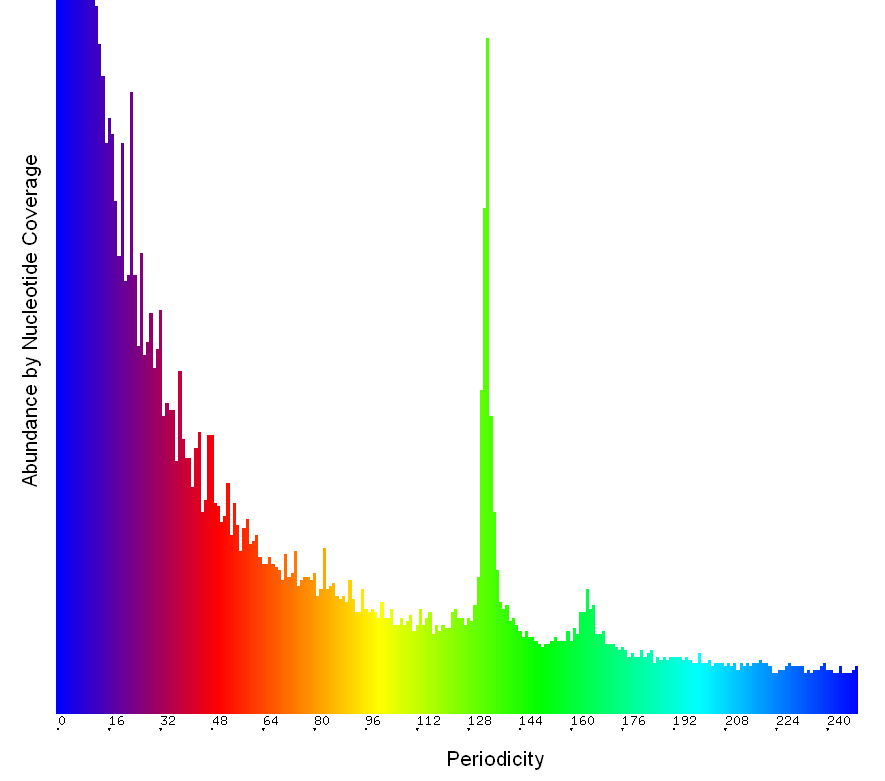

Supplement: Additional file 2 — Histogram of the Repeat Overview pixel hue. This histogram is taken from Human Chromosome 4 as a typical example. There is a very significant peak at 135 which is caused by the di-mer structure of the Alu repeat. Next to it is a much smaller peak centered at 171 caused by the centromere alpha-satellite repeat. Much of the centromere is unsequenced so the number of copies is under-represented. When selecting the 'best frequency' the Repeat Overview starts at offset 1 and all future values must beat the previous by 10%. This is to ensure that it does not display multiples of repeats and also causes the graph to favor shorter repeats. [file 1471-2105-10-452-S2.TIFF]

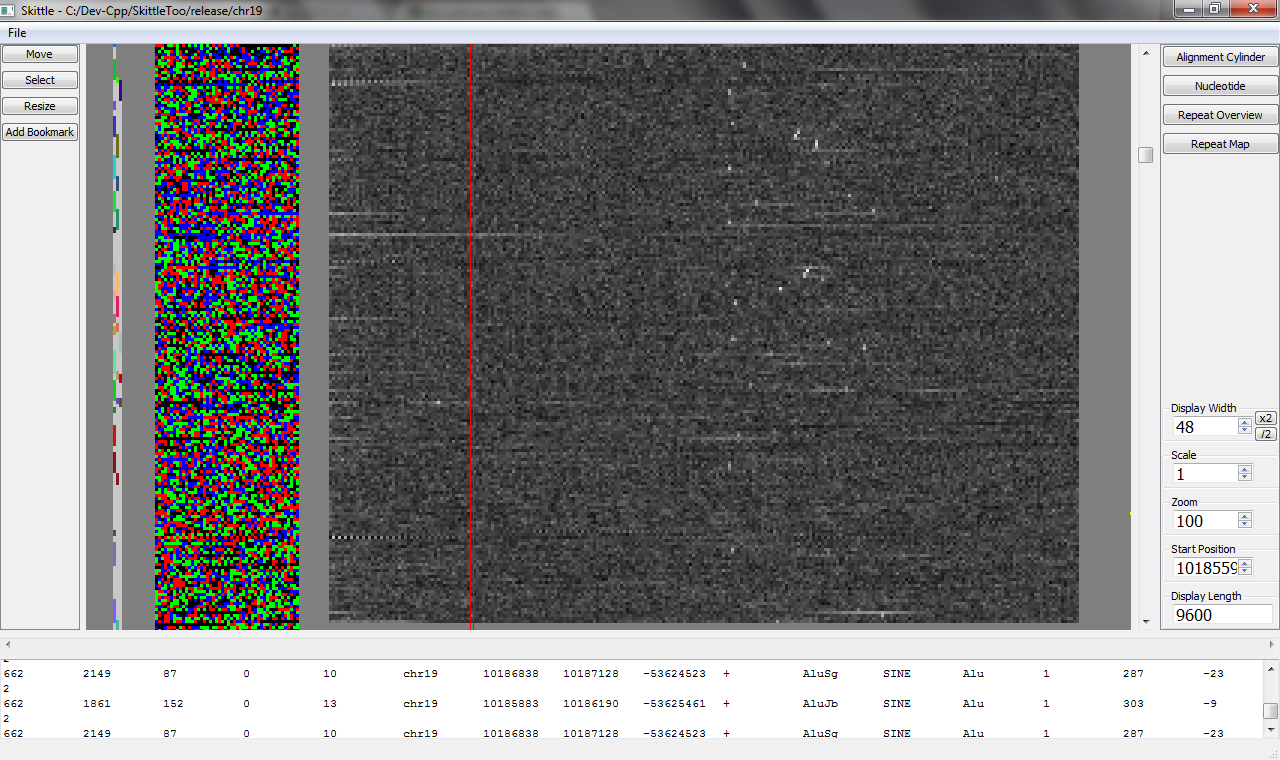

Supplement: Additional file 3 — Alu repeats in Skittle. Alu repeat annotations on Human chromosome 19 (left), shown next to the Nucleotide Display and Repeat Map. This annotation track is based upon RepeatMasker. As seen, this section is rich in Alu SINE repeat annotations, which correlate to pixels seen in the Repeat Map around 135 bp, and which reflect the internal repeat structure seen within most Alus. The Annotation Display appears as three lines in this image because annotation entries often overlap each other. [file 1471-2105-10-452-S3.TIFF]

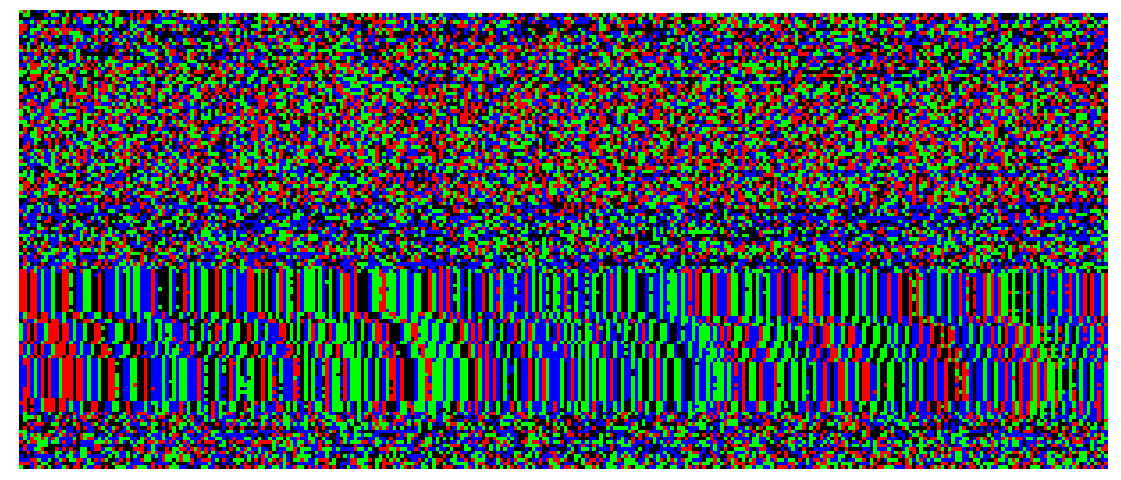

Supplement: Additional file 4 — Large repeats in Drosophila genome. This tandem repeat is found in Drosophila melanogaster chromosome 2L. Its monomer length is 306 bp. The pattern starts at nucleotide #4,533,478. [file 1471-2105-10-452-S4.TIFF]

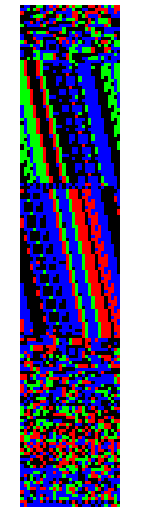

Supplement: Additional file 5 — Reverse Complement Repeat. Drosophila melanogaster: The top and bottom of this tandem repeat are actually reverse complementary sequences, making it a palindrome. Using Skittle, this repeat has been observed on multiple chromosomes. Chr2L: #21020819, Chr2R: none, Chr3L: #8013350, #20815000, #22737200, Chr3R: #17435800, #18277280, Chr4: none, ChrX: #2964690, #3621290, #11539710, #19251467, #21924980. [file 1471-2105-10-452-S5.TIFF]

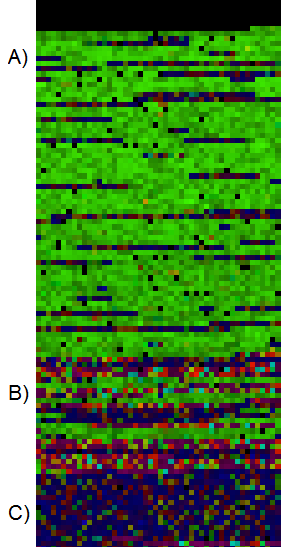

Supplement: Additional file 6 — Repeat Overview of Alternating Repeats. Repeat Overview of a section of Chromosome 19 just below the unsequenced centromeres (black). A) The bright green pixels are alpha-satellite. The mixed dark purple and blue pixels represent areas with no tandem repeats alternating with the alpha-satellite repeat. B) The 35-mer repeat shows up in bright purple and orange. C) The darker colored region at the bottom is where unique, non-repeating DNA resumes after the centromere. Each pixel represents 170 nucleotides. [file 1471-2105-10-452-S6.TIFF]

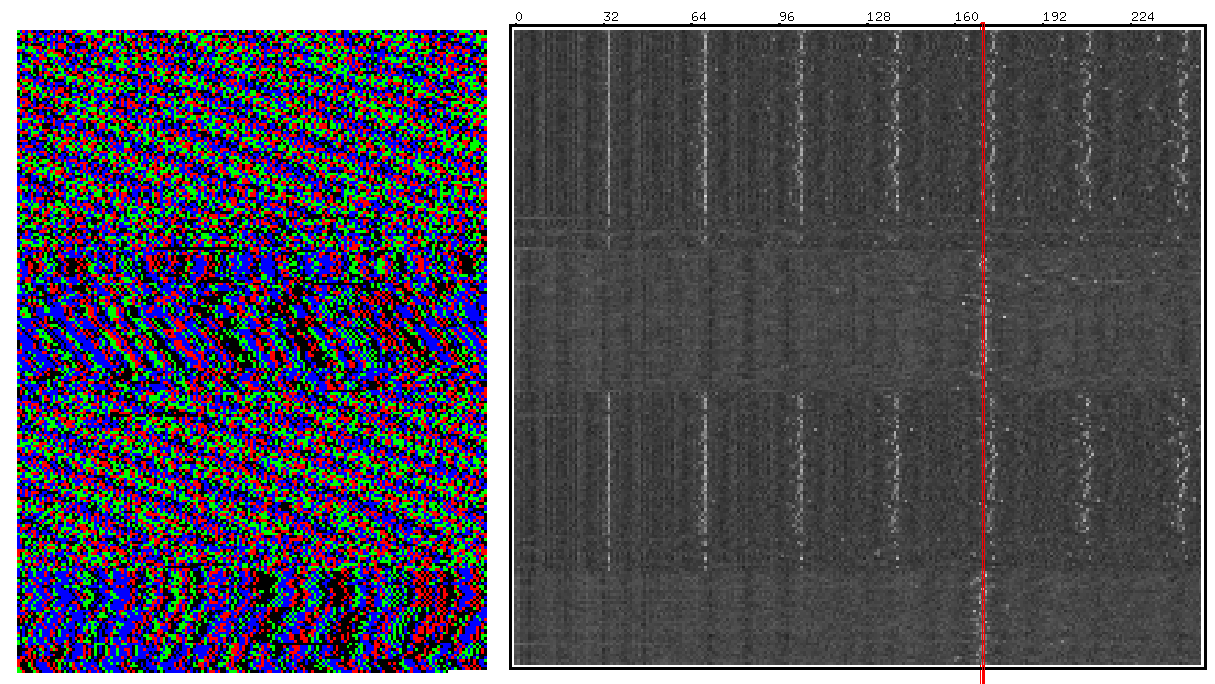

Supplement: Additional file 7 — Nucleotide Display of Alternating Repeats. Alternating 171 and 35 repeats on Human Chromosome 19. These repeats often occur together. [file 1471-2105-10-452-S7.TIFF]

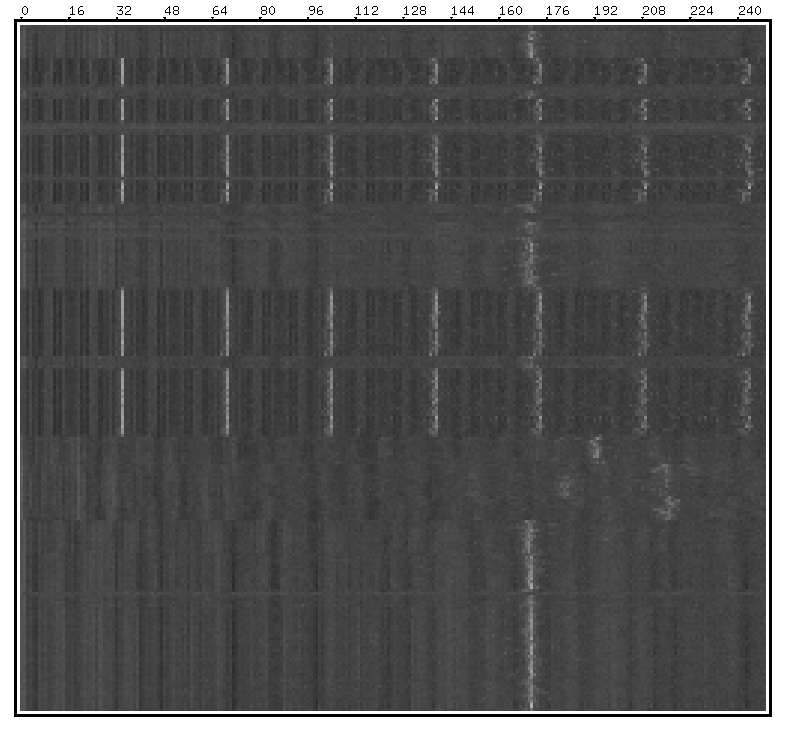

Supplement: Additional file 8 — Repeat Map of Alternating Repeats. Repeat Map showing another example of alternating repeats on Human Chromosome X. Each pixel row of the Repeat Map represents the local alignment of 1380 nucleotides. The map covers over 220,000 nucleotides. The repeats alternate between 5 different patterns: 171, 35, 171, 35, 171, 35, 171, 35, 193, 217, 183/217, 217, 171. This is immediately followed by the unsequenced centromere. [file 1471-2105-10-452-S8.TIFF]

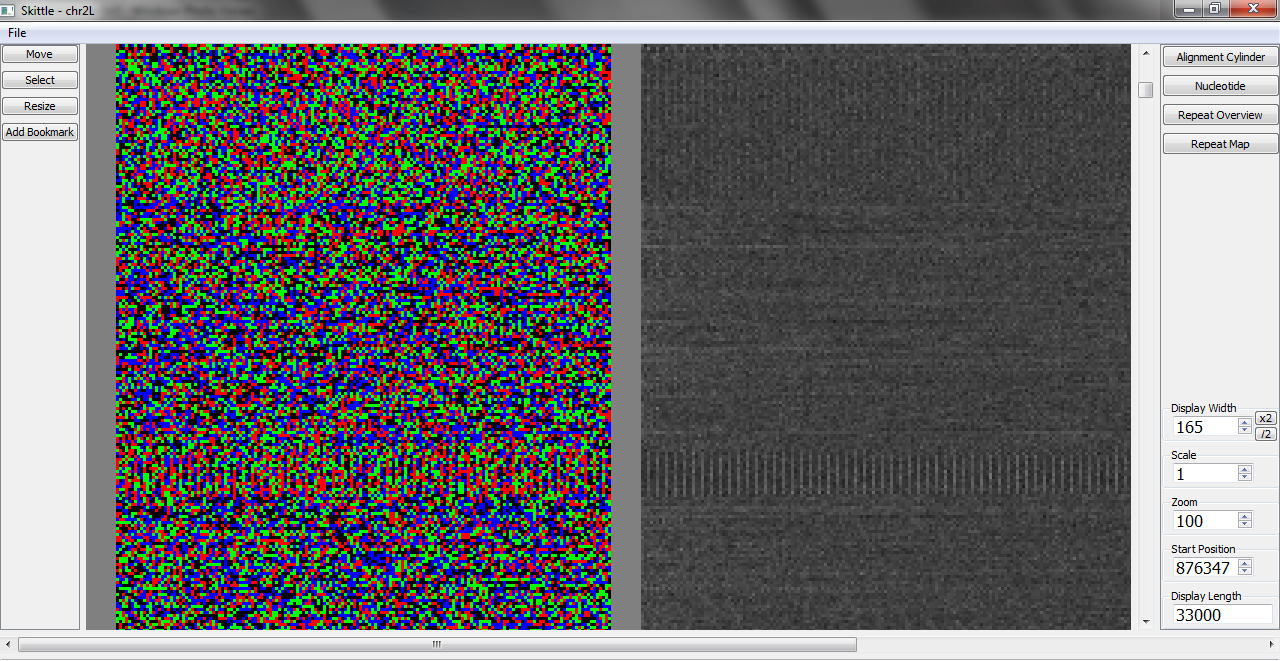

Supplement: Additional file 9 — Genes in Drosophila. Example of visible genes in Drosophila melanogaster chromosome 2L. Exons are accompanied by a faint, though recognizable, 3-mer frequency pattern caused by the codon bias of the organism. Potential exons can be visually identified using Skittle without any other knowledge. In the Human genome, exons are sometimes also visually recognizable because of a sudden change in nucleotide bias towards GC rich content (Additional File 1). [file 1471-2105-10-452-S9.TIFF]

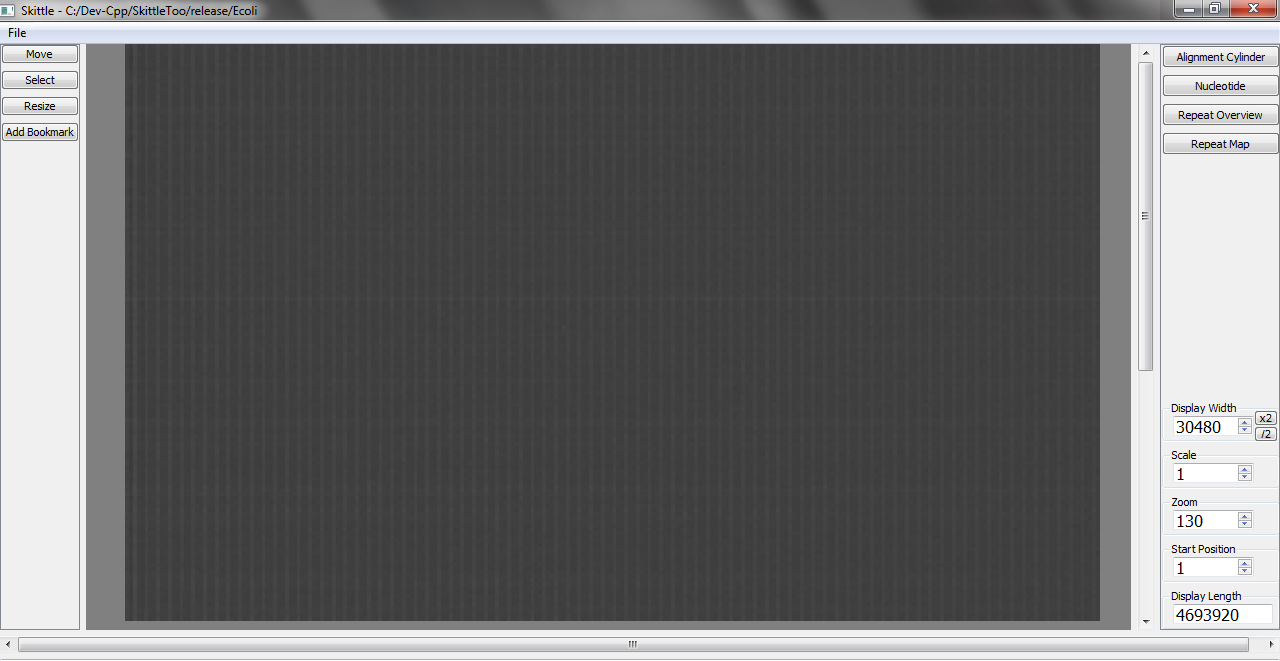

Supplement: Additional file 10 — Repeat Map of E. coli. The Repeat Map of the whole Escherichia coli genome reveals the 3-mer frequency pattern characteristic of exons. The majority of this genome sequence is protein coding, making it a better example than the human genome. The visible pixel pattern is dark, dark, light, which repeats every 3 pixels and is due to codon bias. This is an average of all local alignments ranging from offset 1 to 250, so it effectively negates the fact the individual exons exist in different reading frames. [file 1471-2105-10-452-S10.TIFF]
